# Trace metals in transboundary (India–Myanmar–Bangladesh) anadromous fish *Tenualosa ilisha* and its consequences on human health

**DOI:** 10.1038/s41598-023-47142-4

**Published:** 2023-11-15

**Authors:** Afsana Parvin, Md Kamal Hossain, Afroza Parvin, M. Belal Hossain, Md Aftab Ali Shaikh, Mohammad Moniruzzaman, Badhan Saha, Priyanka Dey Suchi, Fahima Islam, Takaomi Arai

**Affiliations:** 1 Soil and Environment Research Laboratories, BCSIR Laboratories Dhaka, Bangladesh Council of Scientific and Industrial Research, Dr. Qudrat‑I‑Khuda Road, Dhanmondi, Dhaka, 1205 Bangladesh; 2 Cental Analytical Research Facilities (CARF), Bangladesh Council of Scientific and Industrial Research, Dr. Qudrat‑I‑Khuda Road, Dhanmondi, Dhaka, 1205 Bangladesh; 3 School of Engineering and Built Environment, Griffith University, Brisbane, QLD Australia; 4 Bangladesh Council of Scientific and Industrial Research and Department of Chemistry, Dhaka University, Dhaka, 1000 Bangladesh; 5 Environmental and Life Science Programme, Faculty of Science, Universiti Brunei Darussalam, Gadong, Brunei Darussalam

**Keywords:** Environmental sciences, Natural hazards

## Abstract

Hilsa shad (*Tenualosa ilisha,* Hamilton, 1822), the highly coveted table fish within the Indian subcontinent, is Bangladesh's most significant single-species fishery. To assess the risk that toxic metals pose to human health, certain health risk indices—estimated daily intake (EDI), target hazard quotient (THQ), total target hazard quotient (TTHQ), and target cancer risk (TR)—were calculated. The hierarchy of toxic metals (µg/g-ww) in Hilsa shad of the bay showed as Zn (13.64 ± 2.18) > Fe (9.25 ± 1.47) > Mn (2.98 ± 0.75) > Cu (0.57 ± 0.18) > Cr (0.23 ± 0.06) > Pb (0.22 ± 0.04) > As (0.08 ± 0.02) > Ni (0.06 ± 0.02) > Co (0.04 ± 0.01) > Cd (0.01 ± 0.003) in the wet season and Zn (11.45 ± 1.97) > Fe (10.51 ± 1.38) > Mn (3.80 ± 0.75) > Cu (0.73 ± 0.17) > Pb (0.30 ± 0.03) > Cr (0.20 ± 0.05) > As (0.09 ± 0.01) > Ni (0.08 ± 0.02) > Co (0.07 ± 0.02) > Cd (0.02 ± 0.004) in the dry season. The EDI of all the examined trace metals indicated no risk to human health from consuming Hilsa fish. The estimation of THQ and TTHQ suggested that the ingestion of both individual and combined trace metals through Hilsa shad consumption was safe from the perspective of human health. Also, there was no evidence of carcinogenic risk for consumers based on the evaluation of the TR value of metals (As, Pb, Cd, and Ni) due to Hilsa shad consumption.

## Introduction

The coast of Bengal as a dumping ground for barrels of toxic chemical waste has harmful effects on wildlife and potential risks to humans, a potential link between exposure to the chemicals and breast cancer, as well as reproductive problems^1^. Recent evidence suggests that tens of thousands of barrels of waste are disposed of off the Bay of Bengal coastline^1,2^ and the chemicals are still spread across a vast stretch of the seafloor. In addition, river contaminants are hastening the pollution of coastal waterways, which could contaminate the Hilsa shad fish along with other biotas, endangering human health via fish and seafood intake^3^. Trace metals are non-biodegradable in nature, and aquatic organisms and other life forms may encounter rapid accumulation of these trace metals from nature^4,5^. Trace metals exhibit a tendency to accumulate in the gills at higher concentrations compared to the muscles and are additionally retained in the liver as a metallothioneins group^6,7^. Chronic ingestion of trace metal-contaminated fish can result in serious human health issues, including cancer, heart disease, ulcers, asthma, cardiovascular disorders, and nervous system disorders^8–11^.

The transboundary fish is generally found in Bangladeshi rivers and seas (60% of the overall catch), subsequently ahead of Myanmar (with a contribution of around 20%) and India (accounting for approximately 15%), with the rest 5% coming from countries nearby^6^. Importantly, this valuable fish is found in nearly all major riverine habitats in Bangladesh and associated estuaries along with the Bay of Bengal^6,12^. Interestingly, during spawning, most of the anadromous Hilsa stock of the Northern Bay of Bengal (NBOB) migrates toward Bangladeshi water bodies^13^. Thus, Hilsa has been declared as Bangladesh’s national fish and geographical indicator (GI)^14^, accounts for 65% of Bangladesh's marine fish capture and 12% of its overall fish production^12^, and also provides a livelihood for 2% of the population and represents 1% of the country’s GDP^13^. The elevated levels of toxic trace metals in Hilsa shad have emerged as a fretting outcome in more recent investigations conducted in Bangladesh^2,6,15^. These researches indicated that certain metal concentrations in Hilsa exceed food safety guidelines and have carcinogenic and non-carcinogenic risks. The intestinal tissue and fins of Hilsa fish have not been utilized as metal exposure indicators. Given the high demand for Hilsa shad, a comprehensive study of hazardous metals' health risks is imperative in Bangladesh.

To the greatest extent of our knowledge, our team was the first to conduct comprehensive benchmark analyses of the metal in several *T. ilisha’s* organs, such as muscles, fins, gills, and intestines, in wet and dry seasons. Moreover, the findings were compared with national and international guidelines. Furthermore, the probable carcinogenic and noncarcinogenic risks on human health for consumption of Bangladesh’s national fish were also calculated.

## Materials and methods

### Study areas

**Figure 1:**
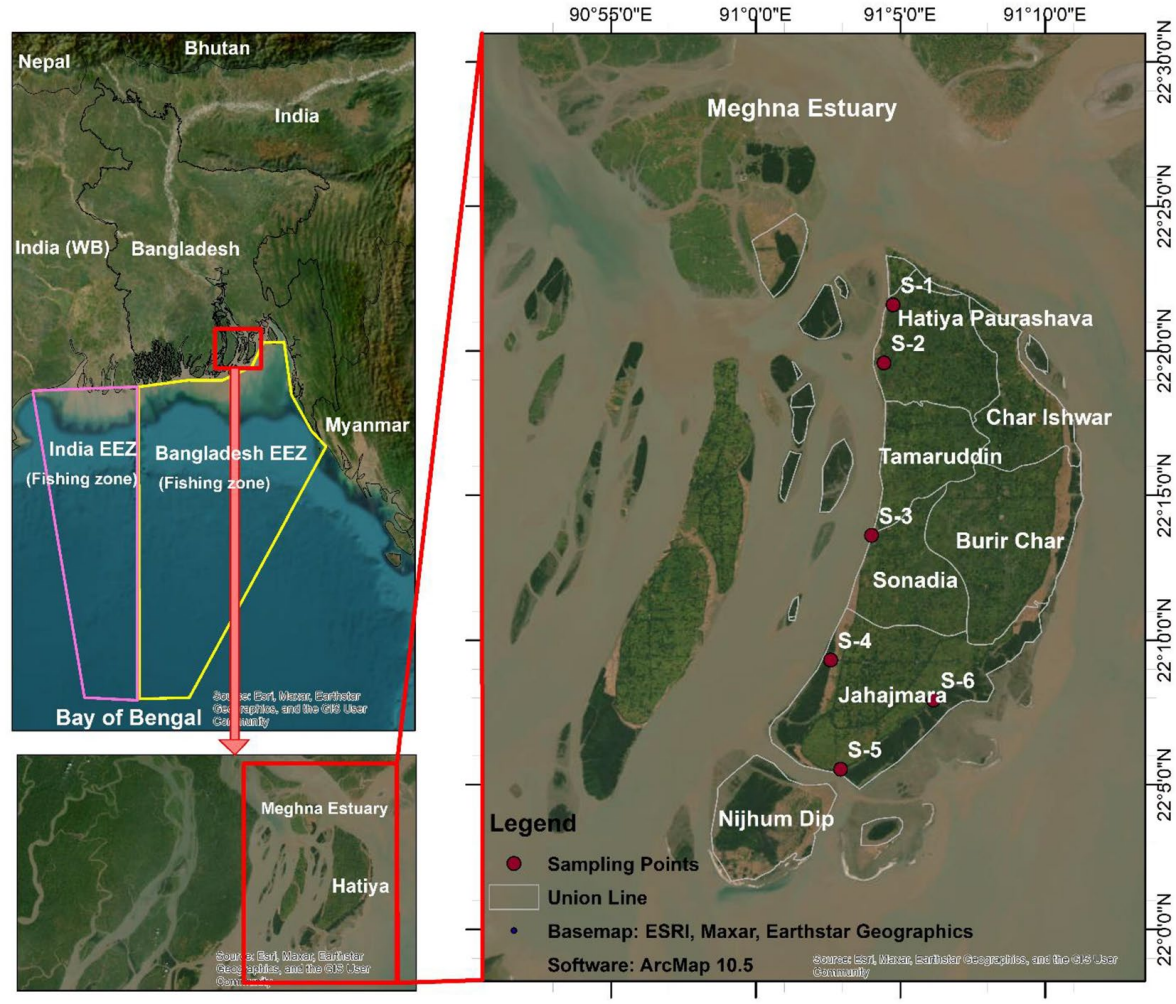
Schematic illustration of the study area (sample collection point) created using software ArcMap version 10.5 (https://desktop.arcgis.com/en/arcmap/latest/get-started/installation-guide/installing-on-your-computer.htm#ESRI_SECTION1_3607D4B46578478B80831714F2A9911A).

Bangladesh, situated within the northeastern Bay of Bengal, has a 710 km long coastline. This coastline is supported by a number of estuaries, including the Meghna and Karnaphully Rivers estuaries, as well as large open water bodies covering an area of 118,813^2^ km^12^. These water bodies are known for their abundance in Hilsa and other fishery resources. The study area was closed to the Meghna Estuary in Hatiya (Fig. 1). The sampling locations and GPS coordinates were presented in supplementary Table S1. Inland rivers carrying industrial effluents from the nation and transboundary nations flow into this estuarine basin where the majority of rivers confluence the Bay of Bengal.

### Sample collection and preparation

For the purpose of investigating the metals (As, Pb, Cd, Cr, Ni, Co, Fe, Mn, Cu, and Zn) dynamics, thirty-two *T. ilisha* specimens with an average length of approximately 35 cm and weight of 650–1000 g were collected from the local fisherman of the selected sample sites as described by Ref.^3^ in two phases: July (wet season) and November (dry season). Following collection, samples of *T. ilisha* were transported to the laboratory by storing them in an ice box. The specimens underwent a rinsing process using deionized water to eliminate surface adherence. Subsequently, inedible components were discarded by means of a sterilized knife. The necessary organs, namely fins, gills, muscles, and intestines, were divided into small pieces and subjected to oven-drying at 70–73 °C for a duration of 48 h until a consistent weight was achieved. Samples were then kept below − 20 °C.

### Analytical methods

#### Sample digestion

0.5 g of dried samples of each organ of the Hilsa shad were precisely weighed using a digital electrical balance, and 10 ml of concentrated nitric acid (68%) and 3 ml of hydrogen peroxide (30%) were added. The samples were then subsequently subjected to microwave digestion (Milestone Start D, Italy) in accordance with the recommended digestion process (AOAC Method 999.10).

#### Elemental analysis by ICPMS and AAS

The process of identifying and measuring metals was conducted utilizing an inductively coupled plasma mass spectrometer (ICP-MS, NexION 2000, Perkin Elmer, USA) and an atomic absorption spectrophotometer (Shimadzu AAS-7000, Japan). The conditions of the instruments for the analysis of metals are provided in Supplementary Table S2. Metals such as As, Pb, Cd, Cr, Co, Ni, Fe, Mn, and Cu were measured using ICPMS, and Zn was analyzed using an atomic absorption spectrophotometer. The limit of detection (LOD) and limit of quantification (LOQ) of each metal are provided in Supplementary Table S3. Calibration curves displaying a correlation coefficient (R^2^) value greater than 0.999 were selected for computing concentrations. The dataset accuracy was confirmed through the utilization of certified reference materials procured from Sigma-Aldrich, Germany. The measurement of the content of trace metals was carried out by calibration using internal standards. Each solution was formulated employing deionized water and analytical-grade reagents.

#### Quality control and assurance

Quality assurance and quality control were maintained by utilizing certified reference material SRM 2976 (Mussel Tissue) of the National Institute of Standards (NIST), duplicate prepared samples, and reagent blanks at random throughout the measurement for each digestion batch. The recovery of the analyzed metals was between 96 and 103%, indicating a good agreement between certified and measured values (Supplementary Table S4).

### Estimation of the health risks to humans

Health risk indices were employed to compute the prospective health hazard linked to the levels of trace elements in the fish, and calculations were made on the basis of wet-weight. Given that the indigenous human population relies on fish as a dietary staple and all the hilsa fish tissues have high C, H, N, and S values (supplementary Table S5), including all fish tissues in the risk assessment, was imperative.

The estimated daily intake (EDI) is a quantitative approach for estimating trace metal’s impact on human health, and it was determined by applying the specific formula^3^:

1 $$\mathrm{EDI }(\mathrm{\mu g}/\mathrm{kg}-\mathrm{bw}/\mathrm{day}) =\frac{\mathrm{FIR}\times \mathrm{C}}{\mathrm{WAB}}$$

The assessment of health risk in relation to its non-carcinogenic impact involved the utilization of the target hazard quotient (THQ) along with the total target hazard quotient (TTHQ), as outlined in Eqs. (2) and (3) respectively^16,17^:

2 $$\mathrm{THQ }=\frac{\mathrm{EFr}\times \mathrm{ ED }\times \mathrm{FIR}\times \mathrm{C}}{\mathrm{RfD }\times \mathrm{BW}\times \mathrm{ TA}}\times 0.001$$

3 $$\mathrm{TTHQ}=\mathrm{THQ }\left(\mathrm{As}\right)+\mathrm{THQ }\left(\mathrm{Pb}\right)+\mathrm{THQ }\left(\mathrm{Cd}\right)+\mathrm{THQ }\left(\mathrm{Cr}\right)+\mathrm{THQ }\left(\mathrm{Ni}\right)+\mathrm{THQ }(\mathrm{CO})+\mathrm{THQ }\left(\mathrm{Fe}\right)+\mathrm{THQ }\left(\mathrm{Mn}\right)+\mathrm{THQ }\left(\mathrm{Zn}\right)+\mathrm{ THQ }\left(\mathrm{Cu}\right)$$

A THQ value less than 1 signifies the absence of a noncarcinogenic risk. When the THQ value is greater than 1, it suggests the presence of a potential health hazard, necessitating the implementation of precautionary measures and safety protocols. A TTHQ value exceeding 1 signifies the presence of a persistent health hazard. According to Ref.^18,19^, 0.1 ≤ TTHQ < 1 = low; 1 ≤ TTHQ < 4 = medium, and TTHQ ≥ 4 = high health risk for individuals.

The target cancer risk (TR) was used in this investigation to calculate the carcinogenic health risks related to Hilsa shad consumption. The risks pertaining to potential carcinogens were quantified by determining the cumulative probability of an individual developing cancer throughout their lifetime of exposure to that particular carcinogen^3,17^. The range of acceptable TR spans from 10^−4^ to 10^−6^. The target cancer risk was calculated employing the specific equation^3,17^:

4 $$\mathrm{TR }=\frac{\mathrm{EFr}\times \mathrm{ ED }\times \mathrm{FIR}\times \mathrm{C}\times \mathrm{CSFo}}{\mathrm{WAB}\times \mathrm{ TA}}\times 0.001$$

The definitions and values of the parameters and variables utilized in the calculations of health risk indices are provided in Table S6 of the supplementary section.

### Statistical analysis

SPSS software (IBM, Version: 25.0, USA) was used for conducting all the statistical analyses of the quantitative data with a predetermined significance level of *p* < 0.05. The graphs were prepared using Microsoft Excel 2013. The estimated heavy metal concentrations, expressed as µg/g-ww, were displayed as mean ± standard deviation. The variations in trace element concentrations among the fish organs were compared using a one-way variance analysis (ANOVA) preceded by Tukey’s post hoc test. A paired sample t-test was conducted to determine whether seasonal fluctuations affected trace metal concentration. The association of metals was investigated by performing a bivariate Pearson’s correlation test. Principal component analysis (PCA) was used for the factor analysis to verify the distribution of the metals.

## Results and discussion

### Trace metal concentrations (µg/g-ww) in *T. ilisha*

The average trace metal concentration found in the organs of Hilsa shad during both seasons is reported in Table 1. Metals content was calculated on the basis of wet-weight. Details regarding individual trace metals are presented and discussed below.

**Table 1:** The concentrations (Mean ± SE, µg/g-ww) of trace metals in fins, muscle, gills, and intestines of *T. ilisha* (N = 128) during the wet and dry season and different international F.S.G. values.

Organ of Hilsa	Sampling season	As	Pb	Cd	Cr	Ni	Co	Fe	Mn	Zn	Cu
Fin	Wet season	0.07 ± 0.01^a^	0.13 ± 0.01^a^	0.00 ± 0.00^a^	0.10 ± 0.00^a^	0.11 ± 0.06^a^	0.04 ± 0.01^a^	8.42 ± 1.79^a^	6.20 ± 1.21^a^	14.08 ± 1.16^a^	0.08 ± 0.01^a^
	Dry season	0.08 ± 0.01^a^	0.26 ± 0.01^ab^	0.01 ± 0.00^a^	0.08 ± 0.02^a^	0.16 ± 0.06^a^	0.06 ± 0.01^a^	9.52 ± 0.06^a^	7.23 ± 0.64^a^	11.69 ± 0.64^a^	0.11 ± 0.01^a^
Muscle	Wet season	0.04 ± 0.01^a^	0.09 ± 0.01^a^	0.01 ± 0.00^a^	0.13 ± 0.01^a^	0.07 ± 0.01^a^	0.01 ± 0.00^b^	3.32 ± 1.64^ab^	3.21 ± 1.27^ab^	4.12 ± 0.01^b^	0.45 ± 0.06^a^
	Dry season	0.05 ± 0.01^b^	0.18 ± 0.01^a^	0.01 ± 0.00^a^	0.10 ± 0.00^a^	0.02 ± 0.00^a^	0.01 ± 0.00^b^	4.38 ± 0.06^b^	0.39 ± 0.01^b^	2.32 ± 0.06^b^	0.51 ± 0.12^b^
Gill	Wet season	0.13 ± 0.06^a^	0.35 ± 0.06^b^	0.02 ± 0.00^ab^	0.16 ± 0.06^a^	0.01 ± 0.01^a^	0.03 ± 0.01^ab^	10.12 ± 2.37^ab^	0.41 ± 0.01^b^	13.09 ± 1.73^a^	0.53 ± 0.13^a^
	Dry season	0.15 ± 0.01^c^	0.40 ± 0.06^b^	0.03 ± 0.01^ab^	0.12 ± 0.00^a^	0.04 ± 0.00^a^	0.04 ± 0.01^ab^	11.01 ± 0.58^a^	4.30 ± 0.06^c^	11.06 ± 0.57^a^	0.66 ± 0.01^b^
Intestine	Wet season	0.07 ± 0.01^a^	0.30 ± 0.04^b^	0.03 ± 0.01^b^	0.53 ± 0.06^b^	0.03 ± 0.01^a^	0.11 ± 0.00^c^	15.11 ± 1.74^b^	2.12 ± 0.59^ab^	23.26 ± 2.90^c^	1.21 ± 0.59^a^
	Dry season	0.09 ± 0.01^a^	0.34 ± 0.06^ab^	0.04 ± 0.01^b^	0.49 ± 0.06^b^	0.09 ± 0.00^a^	0.19 ± 0.00^c^	17.14 ± 0.58^c^	3.28 ± 0.06^c^	20.72 ± 0.06^c^	1.63 ± 0.06^c^
Total Hilsa contain	Wet season	0.08 ± 0.02^α^	0.22 ± 0.04^α^	0.01 ± 0.00^α^	0.23 ± 0.06^α^	0.06 ± 0.02^α^	0.04 ± 0.01^α^	9.25 ± 1.47^α^	2.98 ± 0.75^α^	13.64 ± 2.18^α^	0.57 ± 0.18^α^
	Dry season	0.09 ± 0.01^α^	0.30 ± 0.03^β^	0.02 ± 0.00^α^	0.20 ± 0.05^α^	0.08 ± 0.02^α^	0.07 ± 0.02^β^	10.51 ± 1.38^α^	3.80 ± 0.75^α^	11.45 ± 1.97^β^	0.73 ± 0.17^α^
FSG		n/a	0.3^§^	0.05^§^	1.00^‡^	0.50^†^	n/a	n/a	n/a	50.00^§^	20.0^§^

Here, a, b, c denotes (in column) significant differences among the different organs of *T. ilisha* for each metal (ANOVA, *p* < 0.05); α, β represent (in column) seasonal variations of each metal concentration in *T. ilisha* (paired sample t test, *p* < 0.05);. *SE* standard error; *ww* wet weight; *F.S.G*.: food safety guideline; ^§^^20^, ^‡^^21^, ^†^^22^.

n/a = not available.

A diverse array of trace metal concentrations was observed in four distinct organs of the Hilsa shad. In the context of the wet season, the order of trace elements in the fins from highest to lowest concentration was Zn > Fe > Mn > Pb > Ni > Cr > Cu > As > Co > Cd. In the muscles, the hierarchy was Zn > Fe > Mn > Cu > Cr > Pb > Ni > As > Co > Cd. In the gills, the order was Zn > Fe > Cu > Mn > Pb > Cr > As > Co > Cd > Ni. Lastly, in the intestine, the order of trace metals was Zn > Fe > Mn > Cu > Cr > Pb > Co > As > Ni > Cd. During the dry season, the relative abundance of trace metals in the fins can be observed to follow the sequence: Zn > Fe > Mn > Pb > Ni > Cu > Cr = As > Co > Cd. In the context of muscle tissue, the observed hierarchy was: Fe > Zn > Cu > Mn > Pb > Cr > As > Ni > Co = Cd. The observed order of elements in the gills was: Zn > Fe > Mn > Cu > Cr > Pb > Co > As = Ni > Cd. Finally, within the intestinal system, the order of trace metals in terms of hierarchy was Zn > Fe > Mn > Cu > Pb > Cr > As > Ni > Co > Cd.

### Arsenic (As)

Exposure to arsenic has been associated with various dermatological manifestations, including dermatitis, mild skin keratosis, vasospastic conditions, hyper-keratinization, gross pigmentation, the formation of warts, reduced nerve conduction velocity, and an increased risk of developing lung cancer^8^. The wet season concentrations of arsenic in hilsa were observed to differ from 0.04 µg/g in muscles to 0.13 µg/g in gills, representing the lowest and highest levels, respectively. In the dry season, the gills exhibited the most elevated levels of As (0.15 µg/g-ww), while the muscles displayed the least (0.05 µg/g-ww), as indicated in Table 1. As content in fishes of the southeastern region of Spain was reported to vary between 0.39 and 12.58 µg/g ww^23^. Earlier investigations found that Bangladeshi fish species have arsenic values that differ from 0.76 to 13 µg/g-ww^3^ and 1.01 to 15.2 µg/g-dw^24^.

### Lead (Pb)

Non-essential trace metal lead (Pb) has a number of harmful health consequences, particularly neurotoxicity and nephrotoxicity^25^. *T. ilisha's* gills and muscles displayed the maximum (0.35 µg/g), and minimum (0.09 µg/g) Pb contents during the wet season (Table 1). In the dry season, the gills exhibited the maximum Pb levels (0.40 µg/g), preceded by the intestines, fin, and muscle (0.34, 0.26, and 0.18 µg/g), respectively. The occurrence of elevated levels of Pb in the fish gills was also documented by Ref.^26^. Furthermore, studies have documented varying levels of Pb in Hilsa shad organs ranging from 0.10 to 1.03 mg/kg ww^2^. The levels of Pb in the examined *T. ilisha* samples exceeded the recommended threshold (0.30 µg/g) for human consumption^27^.

### Cadmium (Cd)

Cadmium, an element known for its high toxicity, has the potential to induce severe poisoning even at concentrations as low as 1 µg/g^28,29^. As per the findings of Ref.^30^, Cd accumulation in the human tissues can result in cancer as well as impact the hepatic, pulmonary, renal, skeletal, and reproductive systems. Cadmium builds up in different fish organs and has a high tendency toward bio-concentration. As indicated in Table 1, the intestine displayed the maximum Cd content (0.03 µg/g) among the studied fish organs in the wet season. This was preceded by the gills (0.02 µg/g), muscles (0.01 µg/g), and fins (not detected), respectively. In the dry season, *T. ilisha's* fins had the lowest Cd content (0.01), while its intestines contained the maximum Cd content (0.04 µg/g) (Table 1). Certain commercial fishes from the coastal area of Bangladesh have been found to contain Cd levels varying from 0.03 to 0.08 µg/g-ww^3^. In the current investigation, all fish species were found to contain cadmium levels below the threshold of 0.05 µg/g as stipulated by Ref.^20^. However, cadmium accumulation in fish over an extended period may harm human health.

### Chromium (Cr)

Chromium is a necessary component of a healthy diet as it is crucially involved in the process of lipids and insulin metabolism^31^. Cr was most abundant in the intestine (0.53 and 0.49 µg/g in the wet and dry seasons, respectively), while it was least abundant in the fin (0.10 and 0.08 µg/g in the wet and dry seasons, respectively) (Table 1). Chromium levels span from 6.92 to 12.23 µg/g in fishes of the Dhaleshwari River, Bangladesh^32^. According to our estimated value, the concentration of Cr in *T. ilisha* was below the food safety guideline values set by Ref.^21^.

### Nickel (Ni)

Although incredibly low quantities of nickel exist in the environment, it can nevertheless lead to various respiratory health complications, including pulmonary inflammation, emphysema, and cancer^33^. The maximum level of Ni was recorded in the fin (0.11 and 0.16 µg/g-ww in the wet and dry seasons respectively), which is below the dietary standards of 0.5 µg/g-ww proposed by WHO. In comparison, the least Ni content was noted in gill (0.01 µg/g-ww) during the wet season and in muscle (0.02 µg/g-ww) during the dry season (Table 1). According to the literature, Ni concentration in fish species from Iskenderun Bay ranged from 0.11 to 12.88 µg/g ww^34^. A daily intake of 100–300 µg/g bw/day of nickel has been suggested by Ref.^35^.

### Cobalt (Co)

Cobalt (Co) affects people in two different ways. Co is a minor component required for the synthesis of vitamin B12. However, prolonged exposure could be harmful to human health^36^. The minimum and maximum levels of cobalt were 0.01 µg/g in muscles and 0.11 µg/g in the intestine during the wet season and 0.01 µg/g in muscles and 0.19 µg/g in the intestine during the dry season, respectively (Table 1).

### Iron (Fe)

The highest Fe content (15.11 and 17.14 µg/g in the wet and dry seasons respectively) observed was in the intestines of *T. ilisha*. The lowest Fe concentration (3.32 and 4.38 µg/g in the wet and dry seasons, respectively) was in the muscles of *T. ilisha* (Table 1). The range of iron contents documented in the literature for fish samples from Iskenderun Bay located in the Northern East Mediterranean Sea, Turkey, was 0.82–27.35 µg/g^34^, and for fish species from the middle Black Sea (Turkey) was 9.52–32.40 µg/g^37^.

### Manganese (Mn)

One crucially important trace element is manganese, which is both a structural component of several enzymes and an active ingredient in their functionalities. For adults, 2–9 mg of Mn per day is recommended by Ref.^35^. During the wet season, the fin contained the greatest Mn concentration (6.20 µg/g-ww), while the gill contained the lowest (0.41 µg/g-ww) (Table 1). In the dry season, the fins exhibited the highest Mn content (7.23 µg/g-ww), preceded by the gills, intestine and muscle (4.30, 3.28 and 0.39 µg/g-ww) respectively (Table 1).

### Zinc (Zn)

For both humans and animals, zinc is an essential trace element. According to^38^, a deficiency of Zn can cause growth retardation, taste loss, and hypogonadism, ultimately leading to reduced fertility. Zn levels in organs of Hilsa shad in two seasons are displayed in Table 1. The most elevated levels of Zn (23.26 and 20.72 µg/g in the wet season and dry season seasons, respectively) were measured in the intestines of Hilsa shad, and the minimum concentration (4.12 and 2.32 µg/g in the wet season and dry season seasons respectively) was observed in the muscles of Hilsa shad (Table 1). Since zinc oxide (ZnO) is frequently used in fish processing factories and fish and shrimp hatcheries for supplying oxygen to fish and shrimp larvae, the high Zn level may be related to these sources. Zn concentration in certain edible fishes of Bangladesh ranged between 42.8 and 418 µg/g^39^. Zn contents in our studied samples were below the acceptable thresholds as per international standards, as indicated in Table 1.

### Copper (Cu)

Despite being a necessary trace metal, a high intake of Cu may develop severe toxicological symptoms. According to Sivaperumal et al. [^38^], copper (Cu) interacts with specific proteins, forming enzymes that serve as catalysts in various physiological functions and hemoglobin synthesis. Nevertheless, acute toxicity from Cu can occur at extremely high concentrations^31^. A number of regulatory agencies have established a defined threshold for the permissible concentration of Cu. During both seasons, the intestine exhibited elevated Cu content (1.21 and 1.63 µg/g in the wet and dry seasons, respectively), and the fin had the lowest concentration of Cu (0.08 and 0.11 µg/g in the wet and dry seasons respectively) (Table 1). Previously, copper levels in fish of the Dhaleshwari River, Bangladesh, tended to range from 5.17 to 9.45 µg/g^32^. Compared to international guidelines, Cu content in investigated samples was lower than the permissible levels (Table 1).

### Seasonal comparison of metal contents in Hilsa shad

**Figure 2:**
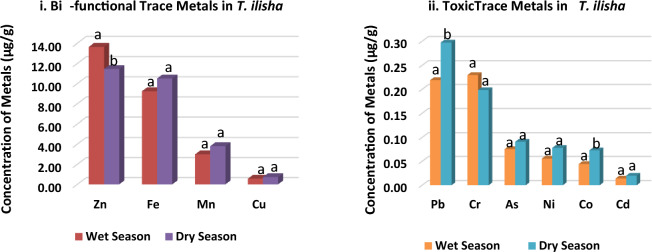
Overall Mean concentration of the trace metals (i: bi-functional trace metals and ii: toxic trace metals) in *T. ilisha* collected from the Bay of Bengal during wet and dry seasons. a, b represents significant seasonal variations of each metal concentration (paired sample t-test (*p* < 0.05).

The concentration of bi-functional and toxic metals present in Hilsa shad across the wet and dry seasons is depicted in Fig. 2i, ii, respectively. These Figs. demonstrated statistically significant seasonal variations in each metal. As per the paired sample t-test (*p* less than 0.05**)**, the wet season Zn level was significantly greater than the dry season Zn content (Fig. 2). On the contrary, Pb and Co contents were significantly higher in the dry season compared to the wet season (Fig. 2). Also, in the dry season, Hilsa samples contained a higher value of Fe (dry season = 10.51 µg/g and wet season = 9.25 µg/g), Cu (dry season = 0.73 µg/g and wet season = 0.57 µg/g), Ni (dry season = 0.08 µg/g and wet season = 0.06 µg/g), As (dry season = 0.09 µg/g and wet season = 0.08 µg/g), Mn (dry season = 3.80 µg/g and wet season = 02.98 µg/g) and Cd (dry season = 0.02 µg/g and wet season = 0.01 µg/g) except Cr (dry season = 0.20 µg/g and wet season = 0.23 µg/g). Globally, numerous studies reported seasonal impacts on metal buildup in fish^16^. In Bangladesh, agricultural effluents, industrial wastes, batteries, and alloys directly enter the open water supply system following the heavy rainfall in monsoon. This might be a significant Cd contamination source in the post-monsoon period. Both Ref.^40^ and^41^ addressed the connection between effluent dumping and the availability of potentially harmful substances in aquatic organisms.

### Association between trace metals in Hilsa shad

Trace metals association differed in wet and dry seasons, as evidenced by Tables 2 and 3, displaying asterisks denoting statistical significance levels of 5% and 1% based on Pearson’s correlation test. A notable positive linear association of As with Pb and Fe occurred during the wet season. The element Pb exhibited a statistically significant positive linear correlation with the elements Cd, Fe, Zn, and Cu. Furthermore, a notable positive linear correlation was observed between the wet season levels of Cd, Co, Cr, Fe, Zn, and Cu. Conversely, a noteworthy positive linear association was observed among the dry season levels of Cd, Co, Cr, Fe, Zn, and Cu. Pb indicated a statistically significant positive association with As, Cd, and Fe in the dry season. Only Ni and Mn displayed a significant positive linear association in both seasons.

**Table 2:** Pearson correlation analysis of metal contents in *T. ilisha* during wet season (stars indicates 2-tailed significance value at 5% level of significance).

Trace metals	Pearson correlations (r)									
	As	Pb	Cd	Cr	Ni	Co	Fe	Mn	Zn	Cu
As	1									
Pb	.749**	1								
Cd	.309	.813**	1							
Cr	.216	.563	.841**	1						
Ni	− .109	− .437	− .407	− .263	1					
Co	.242	.527	.714**	.911**	− .128	1				
Fe	.578*	.788**	.798**	.795**	− .028	.868**	1			
Mn	− .224	− .559	− .442	− .224	.858**	− .079	− .067	1		
Zn	.374	.672*	.781**	.810**	− .066	.909**	.951**	− .019	1	
Cu	.232	.588*	.870**	.829**	− .205	.582*	.657*	− .203	.643*	1

**Correlation is significant at the 0.01 level (2-tailed).

*Correlation is significant at the 0.05 level (2-tailed).

**Table 3:** Pearson correlation analysis of metal contents in *T. ilisha* during dry season (stars indicates 2-tailed significance value at 5% level of significance).

Trace metals	Pearson correlations (r)									
	As	Pb	Cd	Cr	Ni	Co	Fe	Mn	Zn	Cu
As	1									
Pb	.665*	1								
Cd	.432	.834**	1							
Cr	− .007	.377	.789**	1						
Ni	− .010	.054	− .086	.083	1					
Co	.094	.328	.671*	.926**	.329	1				
Fe	.404	.657*	.830**	.831**	.242	.907**	1			
Mn	.378	.330	− .042	− .142	.755**	.153	.318	1		
Zn	.356	.567	.724**	.789**	.388	.925**	.982**	.428	1	
Cu	.109	.363	.804**	.933**	− .170	.826**	.763**	− .335	.688*	1

**Correlation is significant at the 0.01 level (2-tailed).

*Correlation is significant at the 0.05 level (2-tailed).

### Distribution of toxic metals in Hilsa shad of Bay of Bengal by PCA analysis

Table 4 of communalities outlines the variable variance the extracted factors explain. For the purpose of further investigation, only the communality values exceeding 0.5 were considered. Pb, Cr, and Fe accounted for over 90% of the variance compared to other trace metals, accounting for 80–90% of the variance.

**Table 4:** Communality of variance in the amount of trace metals present in *T. ilisha*.

Trace metals	Communalities	
	Initial	Extraction
As	1.000	0.886
Pb	1.000	0.906
Cd	1.000	0.867
Cr	1.000	0.941
Ni	1.000	0.878
Co	1.000	0.883
Fe	1.000	0.968
Mn	1.000	0.897
Zn	1.000	0.834
Cu	1.000	0.833

Extraction method: principal component analysis.

The eigenvalue is a representation of the total number of factors extracted, which should ideally correspond to the number of items that underwent factor analysis. Extracted factors, including their respective eigenvalues, are displayed next. Eigenvalues of more than 1 are needed in order to ascertain the number of distinct factors represented by the variables. According to the findings presented in Table 5, the eigenvalues for the first component (5.53), the second component (2.02), and the third component (1.35) exceed a value of 1, while the eigenvalues for the remaining components are below 1. As a result, three components were represented by the specified set of 10 variables. Moreover, based on the cumulative variance % derived from the extracted sum of squared loadings, the first three components stand for 55.26%, 75.42%, and 88.94% of the variance characteristics, respectively (Table 5). Thus, the features or components pointed out by the aforementioned trace metals were properly represented by three components.

**Table 5:** Total variance explained in the amount of trace metals present in *T. ilisha.*

Component	Initial eigenvalues			Extraction sums of squared loadings			Rotation sums of squared loadings		
	Total	% of Variance	Cumulative %	Total	% of Variance	Cumulative %	Total	% of Variance	Cumulative %
1	5.526	55.264	55.264	5.526	55.264	55.264	4.772	47.716	47.716
2	2.015	20.154	75.418	2.015	20.154	75.418	2.106	21.055	68.772
3	1.352	13.517	88.935	1.352	13.517	88.935	2.016	20.163	88.935
4	0.395	3.953	92.887						
5	0.271	2.705	95.592						
6	0.207	2.071	97.664						
7	0.134	1.344	99.008						
8	0.059	0.591	99.599						
9	0.025	0.255	99.853						
10	0.015	0.147	100.000						

Extraction method: principal component analysis.

**Figure 3:**
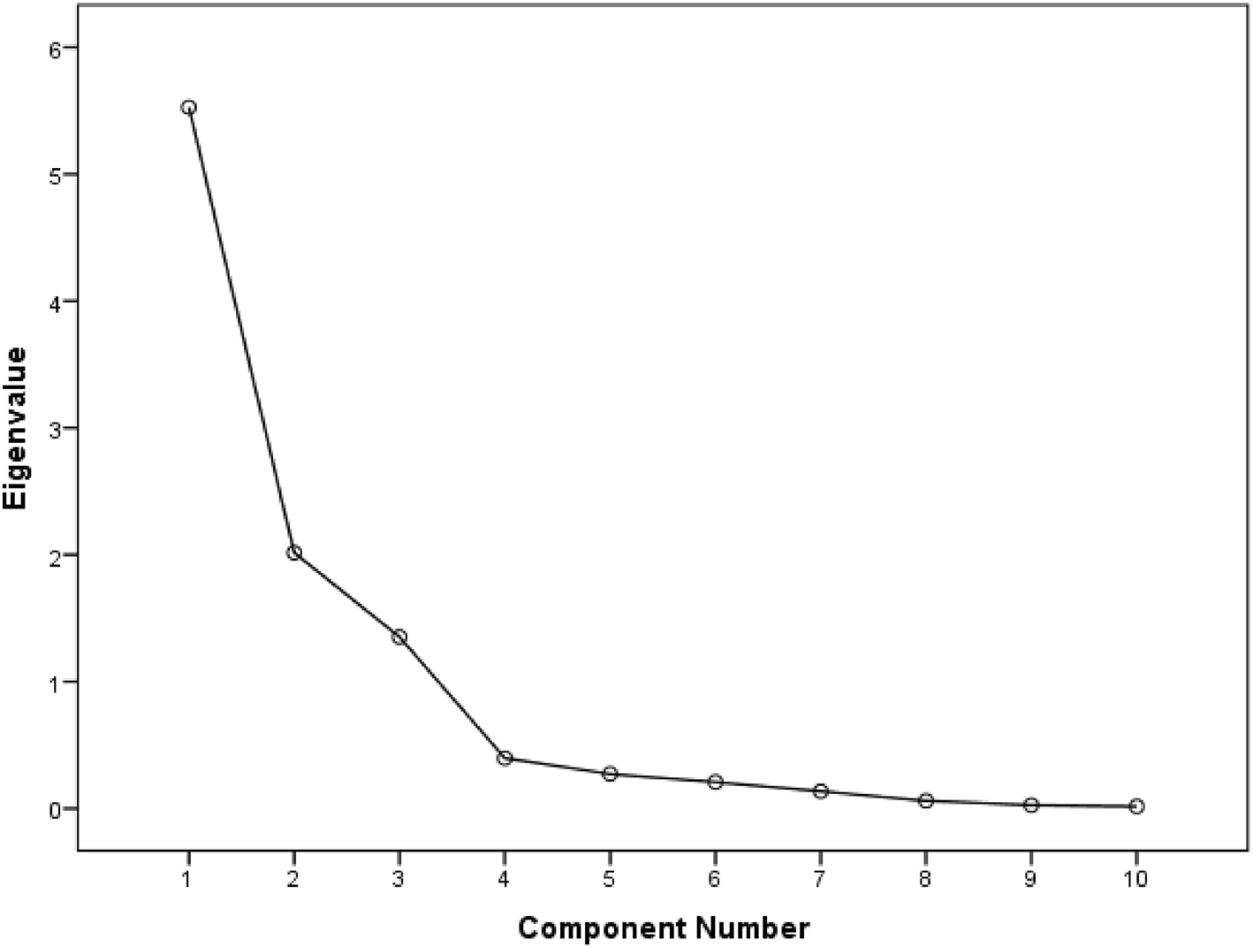
Scree plot of PCA.

The number of factors to retain is decided by utilizing the scree plot, which is prepared by plotting the eigenvalues against each factor. The area of interest is where the curve starts to flatten. Figure 3 illustrates that the curve starts to flatten between factors 3 and 4. Notably, only three factors were kept because factor 4 onwards has an eigenvalue lower than 1.

**Figure 4:**
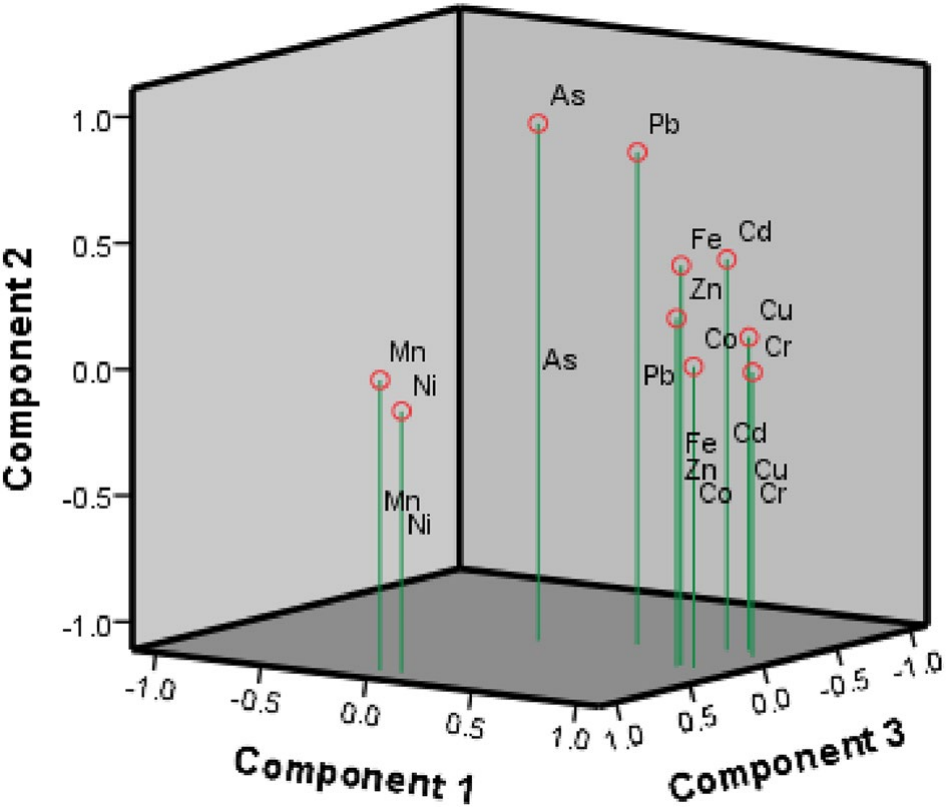
Principal Component Plot in Rotated Space analyzed by scree plot of the characteristic roots (eigenvalues) and component plot in rotated space of trace metals in *T. ilisha*.

The Rotated Component Matrix Table presented the loadings of the variables on the three factors extracted (Table 6). The loading value is directly proportional to the factor's contribution to the variable. Three variables were extracted wherein the 10 items were divided into 3 variables based on the presence of similar responses among the most significant items in component 1, as well as components 2 and 3. The relative locations of trace metals within the first three components are illustrated in Fig. 4.

**Table 6:** Rotated component matrix^a^ of trace elements present in *T. ilisha.*

Trace metals	Component		
	1	2	3
Cr	.959		
Co	.916		
Cu	.868		
Zn	.847		
Fe	.844	.474	
Cd	.796	.438	
As		.938	
Pb	.434	.840	
Mn			.945
Ni			.933

Extraction method: principal component analysis. Three components were extracted; Rotation Method: Varimax with Kaiser Normalization.

^a^Rotation converged in 4 iterations.

### Human health risk assessment

#### Estimated daily intakes (EDIs)

**Figure 5:**
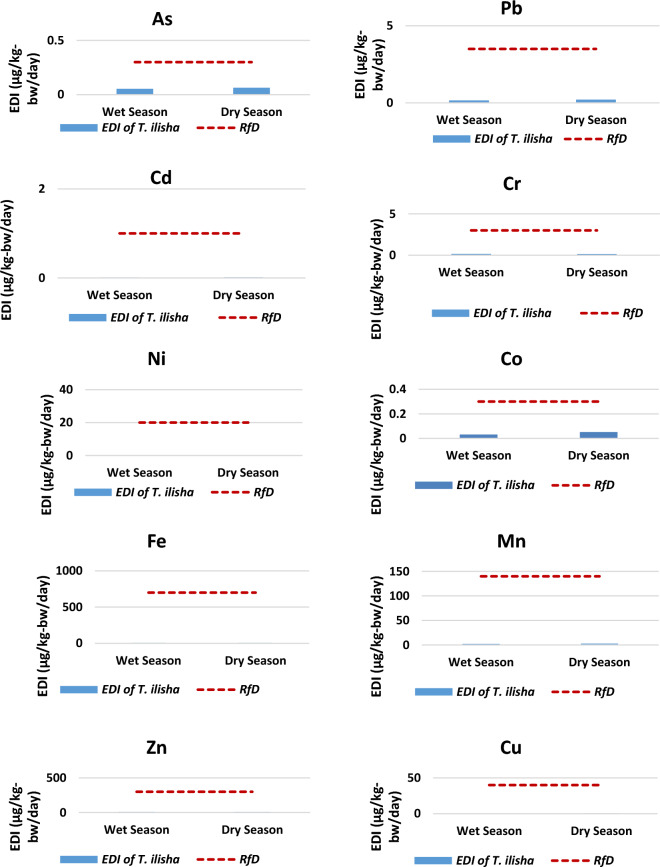
Bar graph showing estimated daily intake (EDI) of selected toxic trace metals from *T. ilisha* consumption by average Bangladeshi People in comparison with oral reference dose (RfD). Red dashed line indicates oral reference dose of individual metals^42,43^.

The values of EDI for the targeted toxic trace metals resulting from the intake of *T. ilisha* by typical Bangladeshi people are displayed in Table 7. The EDIs of metals in *T. ilisha* were assessed through the utilization of the oral reference dose (RfD), which is a quantitative estimation of the level of daily exposure that the human population can endure over their lifetime without a substantial increase in the probability of experiencing adverse consequences. Regarding Hilsa fish consumption, the EDIs of metals in both seasons were lower than the RfDs (Fig. 5). This implies that the typical fish intake would not have had any adverse effects on human health.

**Table 7:** Estimated daily intake (EDI) and Oral Reference Dose (RfD) of trace metals due to consumption of *T. ilisha*.

Metals	Overall mean concentration (μg/g-ww)		Estimated daily intake (EDI) (μg/kg-bw/day)		Oral Reference Dose (RfD)^42,43^
	Wet season	Dry season	Wet season	Dry season	
As	0.076	0.091	0.053	0.064	0.3
Pb	0.219	0.297	0.155	0.210	3.5
Cd	0.014	0.020	0.010	0.014	1
Cr	0.230	0.198	0.162	0.140	3
Ni	0.056	0.078	0.039	0.055	20
Co	0.045	0.073	0.032	0.052	0.3
Fe	9.246	10.514	6.538	7.435	700
Mn	2.983	3.799	2.109	2.686	140
Zn	13.637	11.447	9.643	8.095	300
Cu	0.571	0.728	0.404	0.515	40

#### Noncarcinogenic risk

**Figure 6:**
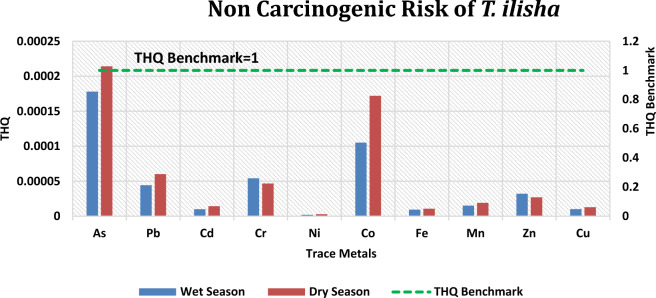
Bar graph depicting estimated target hazard quotients (THQ) of studied metals from *T. ilisha* consumption by average Bangladeshi People. Green dashed line indicates benchmark of non-carcinogenic hazardous condition.

THQs of specific trace metals for typical Bangladeshi consumers of Hilsa shad are depicted in Fig. 6 and Table 8. The average content of individual metals in Hilsa shad was employed to calculate THQ for Bangladesh’s coastal residents. The descending order for the THQ values of metals was As > Co > Cr > Pb > Zn > Mn > Cu > Cd > Fe > Ni in the wet season and As > Co > Pb > Cr > Zn > Mn > Cd > Cu > Fe > Ni in the dry season. According to the findings provided in Table 8, the THQ value for individual metals as well as the TTHQ for combined metals, were found to be below 1 during both seasons, indicating that consuming Hilsa shad would not present significant health risks to consumers.

**Table 8:** THQ, TTHQ, and target cancer risk (TR) of trace metals due to consumption of *T. ilisha.*

Metals	THQ		TTHQ		TR	
	Wet	Dry	Wet	Dry	Wet	Dry
As	0.000178043	0.000214153	0.000460125	0.00057958	8.01195E-05	9.63689E-05
Pb	4.42759E-05	6.0073E-05			1.31721E-06	1.78717E-06
Cd	1.00186E-05	1.4188E-05			6.31174E-05	8.93843E-05
Cr	5.41483E-05	4.67398E-05			–	–
Ni	1.96524E-06	2.76502E-06			6.68182E-05	9.40106E-05
Co	0.000105028	0.000171999			–	–
Fe	9.34054E-06	1.06209E-05			–	–
Mn	1.50678E-05	1.91873E-05			–	–
Zn	3.21445E-05	2.69824E-05			–	–
Cu	1.00929E-05	1.28721E-05			–	–

#### Carcinogenic risk (TR)

**Figure 7:**
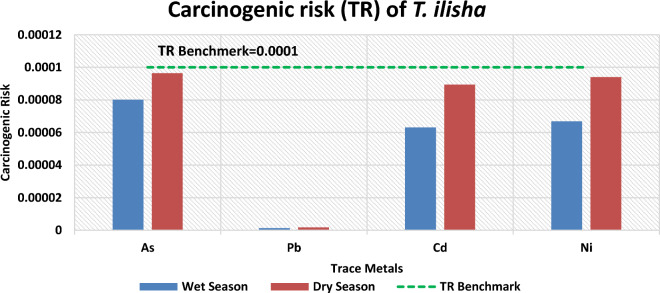
Bar graph depicting estimated carcinogenic risk (TR) of studied metals from *T. ilisha* consumption by average Bangladeshi People. Green dashed line indicates benchmark of carcinogenic risk limit.

The target lifetime carcinogenic risk (TR) of metals (As, Pb, Cd, and Ni) from consuming Hilsa shad is listed in Fig. 7 and Table 8. The estimated values of TR for As, Pb, Cd, and Ni from Hilsa shad consumption during the wet season were 8.01 × 10^–5^, 1.32 × 10^–6^, 6.31 × 10^–5^, and 6.68 × 10^-5^and for the dry season were 9.64 × 10^–5^, 1.79 × 10^–6^, 8.94 × 10^–5^ and 9.40 × 10^–5^ respectively. Cancer risks below 10^−6^ are deemed insignificant, risks exceeding 10^−4^ are generally deemed unacceptable, and risks falling between 10^−6^ and 10^−4^ are typically considered acceptable^44^. The estimated values of TR for As, Pb, Cd, and Ni in both seasons were within 10^−4^–10^−6^, posing a tolerable cancer-triggering risk. Figure 7 clearly illustrates the actual situation in the study area and verifies the tolerable health issues from Hilsa shad consumption. This condition was also corroborated by Ref.^6^, who found no evidence of carcinogenic risk for consumers, with the exception of the largest-sized gills for children. Similarly, Ref.^2^ worked on Hilsa shad and found an acceptable TR value for Pb (4.42 × 10^–6^) and an unacceptable TR value for As (6.63 × 10^–4^).

However, the potential health hazards that individuals may face as a result of consuming metal-contaminated Hilsa shad should not be disregarded. Additionally, not all potential sources of trace metal exposure, like consuming other contaminated foods and inhalation of dust, were considered in the present investigation. Therefore, continual assessment of these toxic trace metals in all food items is required to determine whether there is an imminent health risk to the study area.

## Conclusions

The provision of nutritious and secure protein sources to consumers and economic growth are both of the utmost importance nowadays. The goal of the current study was to establish a thorough understanding of the numerous trace metal levels in organs of the highly lucrative Hilsa shad of Bangladesh during wet and dry seasons and ascertain any potential risks related to the intake of this particular fish species. Investigation results unveiled notable variations in the levels of trace element accumulation within the tissues of Hilsa shad. Though consuming Hilsa shad during both seasons posed no risk to consumers, as determined by the carcinogenic and non-carcinogenic risk assessment, the high dietary metal intake should not be ignored, and attention should be paid to finding an integrated solution to this issue. For these, examining metal bioaccumulation patterns under laboratory conditions, wide-scale repeated sampling in different seasons, and precautionary actions are recommended as future perspectives.

### Supplementary Information

Supplementary Information.

## Data Availability

The datasets used and/or analysed during the current study available from the corresponding author on reasonable request.
