# Supplementary material for: Trace metals in transboundary (India–Myanmar–Bangladesh) anadromous fish Tenualosa ilisha and its consequences on human health
Source: Sci Rep. 2023 Nov 15;13:19978. doi: 10.1038/s41598-023-47142-4 (PMC10651861; doi:10.1038/s41598-023-47142-4)
Supplement: Supplementary file 1 — Supplementary Information. [file 41598_2023_47142_MOESM1_ESM.docx]

**Trace metals in transboundary (India-Myanmar-Bangladesh) anadromous fish *Tenualosa ilisha* and its consequences on human health**

Afsana Parvin^a^, Md Kamal Hossain^a,b^**^*^** Afroza Parvin^a^, Md Belal Hossain^c^, Fahima Islam^a^, Md Aftab Ali Shaikh ^a,b,d,^,Takaomi Arai^e^, Mohammad Moniruzzaman^a,b^, Badhan Saha^a^, and Priyanka Dey Suchi^a^

^a^Soil and Environment Research Laboratories, BCSIR Laboratories Dhaka, Bangladesh Council of Scientific and Industrial Research, Dr. Qudrat‑I‑Khuda Road, Dhanmondi, Dhaka 1205, Bangladesh

^b^Cental Analytical Research Facilities (CARF), Bangladesh Council of Scientific and Industrial Research, Dr. Qudrat‑I‑Khuda Road, Dhanmondi, Dhaka 1205, Bangladesh.

^c^ School of Engineering and Built Environment, Griffith University QLD, Australia

^d^Bangladesh Council of Scientific and Industrial Research and Department of Chemistry, Dhaka University, Dhaka-1000, Bangladesh

^e^ Environmental and Life Science Programme, Universiti Brunei Darussalam Gadong,Brunei, Darussalam

^*^Corresponding Author email: [kamalhossain@bcsir.gov.bd](mailto:kamalhossain@bcsir.gov.bd); [kamalbcsir@gmail.com](mailto:kamalbcsir@gmail.com)

Table S1 Details of the sampling locations

| Sampling Sites | Lattitude | Longitude | Station name |
| --- | --- | --- | --- |
| S-1 | 22.35982 | 91.07927 | Dasher Hat |
| S-2 | 22.32639 | 91.07396 | Bhairab Bazar |
| S-3 | 22.22705 | 91.06681 | Fishery Ghat Char Changa Bazar |
| S-4 | 22.15519 | 91.04318 | Katakhali Ghat |
| S-5 | 22.09227 | 91.04897 | Moktaria Ghat |
| S-6 | 22.13216 | 91.10246 | Nimtoli Bazar |

Table S2 Operating Conditions of NexION ICP-MS Instrument and Atomic Absorption Spectrophotometer

| Inductively Coupled Plasma Mass Spectrometer (ICP-MS, NexION 2000, Perkin Elmer, USA) | | Atomic Absorption Spectrophotometer (Shimadzu AAS-7000, Japan) | |
| --- | --- | --- | --- |
| Operating Conditions | Type or Value | Operating Conditions | Type or Value |
| RF Power | 1600 W | Acetylene | 2.0 L/min |
| Plasma Gas Flow | 15 L/min | Air | 15 L/min |
| Auxiliary Gas Flow | 1.2 L/min | Wavelength | 213.9 nm |
| Nebulizer Gas Flow | Optimized for CeO^+^/Ce^+^ < 2.5%; Ce^++^/Ce < 2.5% | Lamp Current | 08 mA |
| Cell Gas | Oxygen, helium | Slithwidth | 0.7 nm |
|  |  | Detection limit | 0.002 mg/L |

Table S3 LOD and LOQ values of analyzed trace metals by ICPMS and AAS

| Element | LOD (µg/L) | LOQ (µg/L) |
| --- | --- | --- |
| As | 0.08 | 0.30 |
| Pb | 0.0035 | 0.013 |
| Cd | 0.005 | 0.04 |
| Cr | 0.005 | 0.019 |
| Ni | 0.005 | 0.041 |
| Co | 0.004 | 0.011 |
| Cu | 0.08 | 0.24 |
| Fe | 0.16 | 0.52 |
| Mn | 0.021 | 0.051 |
| Zn | 0.002 (mg/L) | 0.007 (mg/L) |

Table S4 Heavy metal concentration (mean ± standard deviation) in certified reference material (SRM 2976), n = 3.

| Element | Certified value (mg/kg) | Measured value (mg/kg) | Mean recovery (%) |
| --- | --- | --- | --- |
| As | 13.3 ± 1.8 | 13.43 ± 1.11 | 101 |
| Pb | 1.19 ± 0.18 | 1.23 ± 0.08 | 103 |
| Cd | 0.82 ± 0.16 | 0.84 ± 0.06 | 102 |
| Cr | 0.50 ± 0.16 | 0.51 ± 0.13 | 102 |
| Ni | 0.93 ± 0.12 | 0.90 ± 0.05 | 97 |
| Co | 0.61 ± 0.02 | 0.62 ± 0.06 | 101 |
| Cu | 4.02 ± 0.33 | 4.02 ± 0.23 | 100 |
| Fe | 171.0 ± 4.9 | 164.16 ± 6.7 | 96 |
| Mn | 33 ± 2 | 32.34 ± 1.3 | 98 |
| Zn | 137.0 ± 13 | 132.89 ± 11 | 97 |

Table S5 C, N, H, and S values of Hilsa shad tissues by Elementar Analyzer

| Organs | Carbon (C) % | Nitrogen (N) % | Hydrogen (H) % | Sulphur (S) % | C/N ratio |
| --- | --- | --- | --- | --- | --- |
| Fin | 28.38 | 10.31 | 5.278 | .638 | 2.75 |
| Muscle | 53.70 | 8.71 | 8.786 | .405 | 6.16 |
| Gill | 43.33 | 5.99 | 6.740 | 0.721 | 7.23 |
| Intestine | 47.53 | 9.34 | 7.985 | 1.337 | 5.09 |

Table S6 Parameters used for health risk assessment calculations

| Symbol | Definition | Values | References |
| --- | --- | --- | --- |
| C | Mean metal concentration in fish (µg/g-ww) | Specific | - |
| FIR | Fish Ingestion Rate (g/person/day) | 49.5 for adult | [1] |
| WAB | Average Body Weight | 70 kg for adult | [2] |
| EFr | Exposure Frequency (days per year) | 365 | [3] |
| ED | Exposure Duration (years) | 70 | [3] |
| TA | Average Exposure Time for Non-carcinogens | 365 × ED | [3] |
| RfD | Oral Reference Dose (µg /kg-bw/day) | 0.3, 3.5, 1, 3, 20, 0.3, 700, 140, 300 and 40 for As, Pb, Cd, Cr, Ni, Co, Fe, Mn, Zn, and Cu, respectively | [4,5] |
| CSFo | Oral Carcinogenic Slope Factor (mg/kg/day) | 1.5, 0.0085, 6.3 and 1.7 for As, Pb, Cd, Ni, respectively. | [6,7,8] |

References

1. Bangladesh Bureau of Statistics (BBS). Statistical Pocket Book of Bangladesh. Government of the People’s Republic of Bangladesh, Ministry of Planning, Dhaka (2015).
2. Tayebi, L. & Sobhanardakani, S. Analysis of heavy metal contents and non-carcinogenic health risk assessment through consumption of tilapia fish (Oreochromis niloticus). *Pollution* **6**(1), 59–67 (2020).
3. Maruf, M. A. *et al.* Assessment of Human Health Risks Associated with Heavy Metals Accumulation in the Freshwater Fish *Pangasianodon hypophthalmus* in Bangladesh. *Expo. Health* **13**, 337–359. <https://doi.org/10.1007/s12403-021-00387-8> (2021).
4. United States Environmental Protection Agency (USEPA). Integrated Risk Information System, CRC (2008).
5. Hang, X. S. *et al.* Risk assessment of potentially toxic element pollution in soils and rice (Oryza sativa) in a typical area of the Yangtze River Delta. *Environ. Pollut.* **157**, 2542–2549 (2009).
6. United States Environmental Protection Agency (USEPA). Risk-based concentration table. http://www.epa.gov/reg3 home/risk/human/index (2010).
7. ATSDR. Coronet Industries, Incorporated (a/k/a Borden Feed Phosphate Complex) Plant City, Hillsborough County, Florida; ID: FLD001704741, U.S. Department of Health and Human Services, Division of Health Assessment and Consultation: Atlanta, GA, USA. Available online: <http://www.floridahealth.gov/environmentalhealth/hazardous-waste> sites/_documents/c/coronet011807.pdf (accessed on 5 December 2021). (2005)
8. United States Environmental Protection Agency (USEPA). Regional Screening Level (RSL) Summery Table. Washington, DC, USA. Available online: https://epa prgs.ornl. gov/chemicals/download/master _sl_table _run _JUN 2011.pdf (accessed on 5 December 2021) (2011).
